# Ex vivo hematopoietic stem cell expansion technologies: recent progress, applications, and open questions

**DOI:** 10.1016/j.exphem.2023.12.001

**Published:** 2024-02

**Authors:** Grace A. Meaker, Adam C. Wilkinson

**Affiliations:** MRC Weatherall Institute of Molecular Medicine, University of Oxford, Oxford, UK

## Abstract

Hematopoietic stem cells (HSCs) are a rare but potent cell type that support life-long hematopoiesis and stably regenerate the entire blood and immune system following transplantation. HSC transplantation represents a mainstay treatment for various diseases of the blood and immune systems. The ex vivo expansion and manipulation of HSCs therefore represents an important approach to ask biological questions in experimental hematology and to help improve clinical HSC transplantation therapies. However, it has remained challenging to expand transplantable HSCs ex vivo. This review summarizes recent progress in ex vivo HSC expansion technologies and their applications to biological and clinical problems and discusses current questions in the field.

## Highlights

• Hematopoietic stem cell expansion is important for basic research and clinical therapies
• Recent advances in culture methods now support long-term hematopoietic stem cell expansion
• Current challenges include functional heterogeneity and potential penalties of expansion

Hematopoietic stem cells (HSCs) are a rare but powerful cell type with the role of supporting blood and immune cell production throughout our lives. It is estimated that there are only ∼100,000 active HSCs in adult humans, yet they are responsible for generating ∼90% of the cells in our bodies [1,2]. HSCs can also stably regenerate the entire blood and immune system following transplantation. To achieve these functions, HSCs balance self-renewal to generate more stem cells and differentiation to generate any of the mature blood cell types through a spectrum of intermediate progenitor cells [3]. The mechanisms underlying these biological activities and cell fate decisions remain incompletely understood, at least in part due to the paucity of HSCs and a lack of culture systems to study them ex vivo.

Functional HSCs mainly live in the bone marrow (at a frequency of ∼1:30,000) and the spleen (at a frequency of ∼1:300,000) in adults and reside in the fetal liver, placenta, and umbilical cord blood in the fetus. During steady-state hematopoiesis, HSCs are largely quiescent but enter the cell cycle to symmetrically or asymmetrically self-renew or to differentiate in response to hematopoietic stress or to maintain homeostasis [4]. A delicate homeostatic balance of self-renewal and differentiation is essential. Perturbations in HSC activity have serious consequences, e.g., in the form of leukemias, myelodysplastic syndrome, and bone marrow failure syndromes [5]. Manipulating HSC activity is of interest from a therapeutic perspective, particular in the context of HSC transplantation (HSCT) [6].

HSCT is a curative therapy for many blood disorders, particularly blood cancers and inherited blood diseases (e.g., primary immunodeficiencies). It involves depleting a patient's diseased bone marrow and hematopoietic system by preconditioning (currently achieved by genotoxic chemotherapy and/or radiotherapy) and then regenerating a healthy hematopoietic system by transplantation of HSCs from a healthy donor (allogeneic HSCT) or from their own healthy stem cells (autologous HSCT) [7]. Although bone marrow HSCs can (and are) used in HSCT, HSCs are now most often collected from the peripheral blood following mobilization [6]. An alternative source of donor HSCs for HSCT is umbilical cord blood (UCB). Unfortunately, single UCB units rarely contain sufficient numbers of donor HSCs to achieve engraftment in adults, limiting their current use. However, the use of UCB in HSCT does have several advantages, including having a lower risk of causing graft-versus-host disease (GvHD) [8]. GvHD is a serious complication in which T cells transplanted alongside the HSCs recognize the patient's body as nonself and attack it^7^. It is important to remember that donor T cells also play a key role in long-term disease remission following HSCT because donor T cells also confer a graft-versus-leukemia (GvL) effect.

Despite being used clinically for more than 60 years, HSCT is a still high-risk therapy, and not all patients can access it. Patients who underwent allogeneic HSCT must find a suitable human leukocyte antigen (HLA) matched donor to reduce the risk of GvHD. Unfortunately, 70% of patients do not have a related matched donor accessible [8]. Additionally, even after HLA matching, allogeneic HSCT recipients often require long regimens of immunosuppressive drugs to prevent GvHD, which carries a serious risk of fatal infections [9]. Autologous HSCT avoids the potential for GvHD and can also be combined with gene therapies, for example, to treat inherited blood diseases, such as primary immunodeficiencies and heredity anemias [10]. In both settings, however, preconditioning can cause substantial acute toxicities and result in neutropenia before donor engraftment, which exposes patients to opportunistic infections. Graft rejection and graft failure also represent serious complications associated with HSCT.

Various approaches to improve the safety and efficacy of HSCT are currently being investigated. One approach that has the potential to improve various aspects of HSCT is the ability to transplant larger doses of HSCs through the use of ex vivo expanded HSCs. Unfortunately, it has long remained challenging to maintain and expand functional HSCs ex vivo [11]. Ex vivo HSC culture conditions also have substantial potential to investigate and characterize this important type of stem cell. In this review, we discuss advances in ex vivo HSC culture technology over the last 5 years and summarize how ex vivo HSC expansion can impact scientific research and clinical therapy as well as look to the future with questions for the field which remain unanswered to date.

## RECENT PROGRESS IN EX VIVO HSC EXPANSION CULTURES

HSCs have traditionally been cultured with prosurvival and self-renewal-inducing cytokines in media containing serum albumin (in the form of serum, bovine serum albumin, human serum albumin, or recombinant serum albumin) [11]. Various cytokines and growth factors have been used in these cultures but typically include thrombopoietin (THPO), stem cell factor (SCF), Flt3 ligand (FLT3L), interleukin(IL)-6, and/or IL-11. Unfortunately, these conditions could generally only maintain or weakly expand mouse and human HSCs during short-term (days-week) cultures. HSC expansion could be improved in these conditions when enforced transgene expression (e.g., Hoxb5) is used, as reviewed elsewhere [12]. However, the applications of these transgenic methods are relatively limited compared with transgene-free methods and will therefore not be discussed further here.

Various efforts over the years have aimed to identify novel additives to enhance ex vivo HSC expansion. There has been a particular focus on small molecule inhibitors, growth factors, and nutrients to expand human UCB HSCs to allow single units to be safely used in HSCT. Because these human HSC agonists have been recently reviewed in detail elsewhere 13, 14, 15, this section will focus on two HSC agonists with recent clinical successes. We will also focus on recent preclinical advances in ex vivo HSC expansion using polymers and novel methods to maintain quiescent HSCs ex vivo.

### Nicotinamide Riboside

In a major milestone for the field, the first expanded HSC product was approved by the US Food and Drug Administration (FDA) in 2023. Omisirge (omidubicel-onlv or NiCord) was approved for use in adults with hematologic malignancies following myeloablative condition. Omisirge is a cord blood HSC product expanded using nicotinamide riboside. Nicotinamide is a form of vitamin B3 and was first reported as an HSC agonist in 2012, where it was shown to support HSC expansion through the inhibition of the deacetylase Sirtuin 1 [16]. In xenotransplantation assays, the addition of nicotinamide was shown to increase engraftable cells (defined by a short-term 6-week end point) by ninefold over a 3-week culture while expanding total nucleated cells by ∼500-fold. Interestingly, dietary supplement of nicotinamide has been more recently shown to stimulate hematopoiesis through driving mitochondrial clearance in HSCs [17], suggesting potential additional mechanisms of action.

### UM171

A second HSC agonist, UM171, has also recently shown clinical promise in phase I/II clinical trials [18,19]. UM171 was first identified in 2014 as a human HSC agonist from a large-scale small molecule screen [20]. It was shown to expand long-term engraftable HSCs by ∼30-fold over a 10-day culture period. Although the mechanism of action was initially unclear, UM171 was recently shown to target the histone demethylase LSD1 and the CoREST repressive epigenetic complex for degradation [21,22]. UM171 mediates target protein degradation via the CULLIN3-E3 ubiquitin ligase complex. Additional targets of UM171 have since been identified [23], suggesting a complex mechanism of action. In culture, UM171 is often used in combination with a second small molecule, StemRegenin1 (SR1), an aryl hydrocarbon receptor antagonist that was also identified by small molecule screening [24]. SR1 is also an HSC agonist in cytokine-based cultures (expanding engraftable HSCs by 17-fold) and appears to synergize with UM171. The receptor tyrosine kinase RET ligand GDNF/GFRα1 has also recently been shown to synergize with the UM171/SR1 combination, boosting HSC output by ∼2-fold [25]. These highlight how numerous molecular pathways feed into ex vivo HSC expansion.

#### Polymer Cultures

An alternative strategy to improve expansion conditions has been to modify the base media, such as through the recent use of 3-dimensional zwitterionic hydrogels, which have been used to improve human HSC expansion [26]. Additionally, we recently discovered that the replacement of serum albumin with synthetic polymers, such as polyvinyl alcohol (PVA), could dramatically improve mouse HSC expansion [27]. PVA is generated by the partial hydrolysis of polyvinyl acetate, giving PVA an amphiphilic structure that is thought to partially mimic the functions of albumin, such as stabilization of recombinant cytokines [28]. However, unlike serum albumin it is a chemically synthesized polymer and does not contain biological contaminants that drive HSC differentiation [27]. In combination with optimized concentrations of THPO and SCF and the use of fibronectin-coated plates, mouse HSCs could be expanded for several months, and transplantable HSCs were expanded several hundred-fold [27]. PVA cultures could also expand single HSCs. These cultures were also highly selective; after a month-long culture, transplantable HSCs were retained at a frequency of ∼1:34.

Since PVA was reported in 2019, several modifications to the protocol have been suggested. High molecular weight PVA was shown to further improve expansion [29]. While in initial reports, HSC cultures were initiated with purified long-term HSCs, Ochi et al. [30] found that these PVA culture conditions were sufficiently selective for HSCs to afford expansion from c-Kit+ bone marrow hematopoietic stem and progenitor cells. To further simplify the protocol, fibronectin-coated plates were also swapped for hydrophilic plastic (CellBIND) tissue culture plates. We recently discovered that the use of physoxic (5% oxygen) culture conditions could further improve the selectivity and purity of PVA-based HSC culture conditions, allowing selection and expansion of transplantable HSCs out of whole bone marrow and spleen [31].

Although PVA-based cultures support efficient expansion of mouse HSCs, human HSC growth was more limited [27]. Sakurai et al. [32] recently overcame this limitation by developing a more efficient and chemically defined method for human HSC expansion. THPO and SCF were replaced with the small molecules butyzamide and 740Y-P, respectively, and UM171 was included in the media to inhibit megakaryocyte differentiation. A copolymer of polyvinyl-caprolactam, polyvinyl acetate and polyethylene glycol called Soluplus was also identified as supporting more rapid growth of human HSCs ex vivo than PVA. Together, these media conditions supported ∼70-fold growth of UCB-derived CD34+ HSPCs over 30 days. These cultures could even expand single human HSCs clonally. It is worth highlighting that Soluplus has now also been shown to support mouse HSC expansion [33]. Soluplus appears particularly beneficial for clonal expansion of mouse HSCs. In clonal expansion studies, it is estimated Soluplus can support ∼18,000-fold expansion from a single HSC during a one month culture [33].

### Quiescent Cultures

Although these innovations in *ex vivo* HSC expansion culture conditions are important to yield large numbers of HSCs for clinical applications or experimental assays (discussed below), culture conditions that drive HSCs into proliferation do not completely model in vivo adult HSC behavior under homeostasis where the HSC population is largely quiescent. To better model this state, the Takubo laboratory recently developed hypoxic (1% oxygen) and low-cytokine (SCF and THPO) ex vivo culture condition to support maintenance of quiescent mouse and human HSCs [34]. Additionally, the same group has established methods to genetically edit HSCs in these ex vivo quiescent conditions, expanding the potential applications of this culture system to study HSC quiescence [35]. It will also be interesting to consider how emerging bone marrow organoid technologies [36,37] can be used to grow and study HSCs ex vivo within the context of the bone marrow microenvironment.

## APPLICATIONS OF EX VIVO HSC EXPANSION TECHNOLOGIES

As demonstrated in recent clinical trials, transplantation of allogeneic UCB-derived HSCs expanded nicotinamide [38] or UM171 [18] into patients with hematologic malignancies can improve the safety of HSCT. Compared with transplantation of unmanipulated UCB, Omisirge (nicotinamide-expanded HSCs) was shown to reduce the time to neutrophil recovery from an average of 22–12 days. It also reduced the likelihood of serious infection in the first 100 days post-transplantation from an average of 60%–39%. Similar improvements were seen with UM171-expanded HSC products, with an average neutrophil engraftment time of 9.5 days. Alongside the improved safety of HSCT, these expanded HSC products should also improve patient access to HSCT because more UCBs (with a wider range of HLA combinations) become available for clinical use, increasing the chance of finding a good donor match [39].

The clinical successes discussed above highlight the relevance of expanded HSC products to improve patient treatment option, even when expansion of functional HSCs is relatively small. Once higher rates of HSC expansion can be achieved at a clinical scale, further improvements in the HSCT treatment paradigm should become possible. Some of this potential has already been highlighted in preclinical mouse studies. For example, once we can achieve more robust HSCs, we should be able to use a single UCB (or donor) to provide HSC products for multiple patients. In polymer-based mouse HSC expansion, even single HSCs could expand sufficiently to engraft in multiple recipients [33].

### Nongenotoxic HSCT

Large doses of expanded HSCs may also help to reduce the use of genotoxic preconditioning and its associated toxicities. Preconditioning plays two roles in HSCT: destroying endogenous HSCs (and hematopoiesis) to boost donor HSC engraftment and destroying the patient's immune system to prevent rejection of allogeneic donor cells. PVA-expanded mouse HSCs could robustly engraft in nonconditioned recipient mice, including those genetically modified by lentiviral transduction [27,30]. This was achieved in the autologous setting, as well as in the allogeneic setting for immunodeficient recipients. Although this approach would not be feasible for standard allogeneic HSCT, there could be interesting applications for this type of approach to autologous HSCT gene therapies. Additionally, we recently showed that expanded HSCs could engraft in immune-mismatched mice without genotoxic preconditioning when antibodies were used to block immune rejection. In a Fanconi anemia mouse model, high long-term donor chimerism was achieved when wild-type expanded HSCs were transplanted into anti-CD4 antibody conditioned recipient mice [31].

### HSC Gene Therapy

Alongside the use of ex vivo HSC expansion conditions for allogeneic HSCT, there are also potential applications for autologous HSCT, particularly in the context of gene therapies. The advances in gene editing technology, particular CRISPR, have stimulated efforts to genetically correct a range of genetic blood diseases. CRISPR is a tool which was first discovered as a primitive bacterial immune system and has now been developed into a highly programmable nuclease, suitable for targeted gene disruption, insertions and even tweaking their expression 40, 41, 42, 43. Recent clinical trials using CRISPR-edited HSCs to treat sickle cell disease and β-thalassemia have yielded impressive results [44]. However, time to engraftment was noticeably delayed, with neutrophils only detected after 30–33 days. Additionally, a second HSC gene editing clinical trial (NCT04819841) was recently terminated due to engraftment failure. Improvements in ex vivo HSC culture conditions should help to provide more gene-corrected functional HSCs back to the patient and thereby improve the safety of these exciting gene therapies.

Current gene therapies transplant a mixed bulk population of genetically corrected and noncorrected HSCs. Becker et al. [33] recently demonstrated that efficient ex vivo expansion of single HSCs allows for genetically defined populations of HSCs to be transplanted. This is achieved by sorting and expanding single HSCs following gene editing and then genotyping the clonally-derived cultures before transplantation. Although this may not be necessary for all gene editing strategies, it represents a potentially powerful approach to avoid transplanting HSCs carrying unwanted genetic mutations.

### HSC Genetic Perturbations

The clonal expansion of gene edited HSCs is also likely going to become a very valuable tool for basic research, where the function of HSCs carrying specific a genetic mutation can be investigated. This clonal expansion method may avoid the need to generate transgenic mouse lines to investigate HSC genetic perturbations, saving time and resources. Even without single cell cloning, the genetic modification of HSCs in ex vivo expansion cultures have already been used in basic research and preclinical models [30,45]. These technologies can be readily applied to ask a range of questions about the biology of HSCs and the hematopoietic system or used to generate or treat disease models.

### HSC Genetic Screens

Beyond targeting single genes with CRISPR, the large numbers of HSCs generated by recent expansion culture conditions now afford CRISPR screening to be efficiently performed. This has recently been elegantly demonstrated by Lara-Astiaso et al., [46] who performed a CRISPR knockout screen for 142 chromatin factors for regulators of HSC expansion and differentiation. We have also recently performed large-scale CRISPR knockout screens (targeting ∼7,500 genes) using expanded HSCs which were then transplanted into irradiated recipient mice to identify regulators of hematopoiesis [47]. These approaches may ultimately identify new approaches to further boost HSC expansion ex vivo.

### Reducing Animal Usage

More generally, ex vivo HSC expansion represents a valuable method to generate sufficient numbers of HSCs for many experimental applications. Another important implication of mouse HSC expansion culture systems is the ability to reduce animal usage. With only a few thousand HSCs collectable from a single mouse, molecular assays often require HSCs to be harvested from many donor mice. Cultures that support 100–1,000-fold expansion can therefore dramatically reduce animal usage while at the same time opening up exciting new opportunities for investigation. These reductions not only reduce financial costs but also the ethical costs of research. It is important to note, however, that at this time these in vitro assays are not a replacement. In vivo transplantation assays remain the only way to confirm HSC activity because we currently cannot mimic the complex cellular interactions and responses that HSCs must achieve to stably reconstitute a functional blood system within in vitro systems. In the future, it will be interesting to understand whether these culture systems can also be adapted to quantify HSC activity ex vivo and thereby reduce the current reliance on in vivo HSC transplantation assays. Human HSC expansion conditions are also important for basic research, with the potential for small patient-derived samples to generate sufficient HSC numbers for deep molecular interrogation.

## OPEN QUESTIONS AND CONCLUSIONS

**Figure 1:**
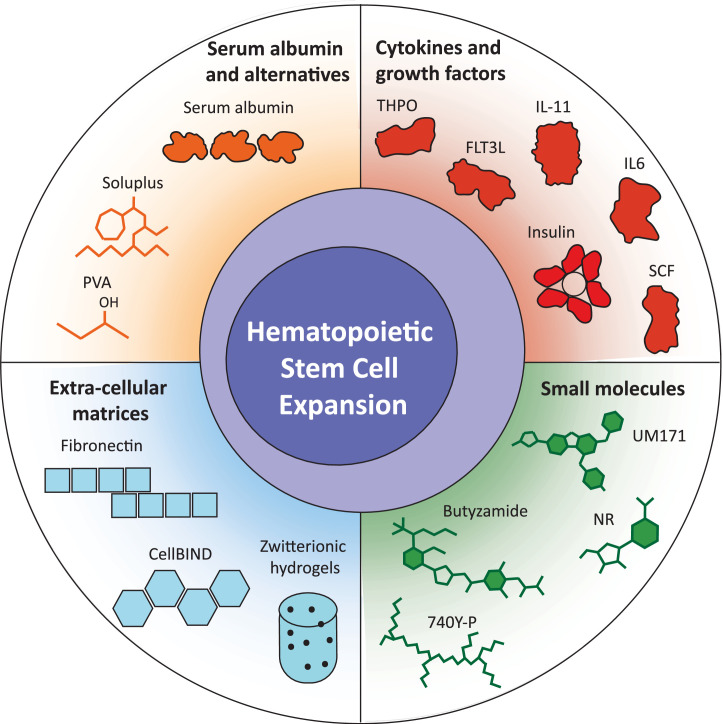
Components supporting ex vivo hematopoietic stem cell expansion cultures. A graphical summary of the culture components recently applied to improve HSC expansion ex vivo. Structures simplified for illustrative purposes.

Technologies underpinning ex vivo HSC expansion are in a phase of rapid development in both the basic biology and clinical contexts (Figure 1). Despite recent technical developments, there are still many unanswered questions that warrant further investigation. Below, we outline current open questions in the field and the progress toward answering them.

### Which Types of HSCs Expand Best?

To date, most research as focused on expanding young adult mouse bone marrow-derived HSCs (due to the common use in in vivo studies) or human UCB-derived HSCs (due to their clinical potential for allogeneic HSC transplantation). However, we know that HSC activity changes with ontogeny and age [48,49]. It remains to be determined how well culture conditions optimized for one HSC type work for other HSC types (and what the expansion potential of these different HSC types are). In particular, less effort has been put into expanding adult human HSCs from bone marrow or mobilized into the peripheral blood [14]. However, the rapidly growing interest in ex vivo HSC gene therapies highlights the importance of being able to efficiently maintain and expand adult human HSCs [50]. Additionally, the generation of transplantable HSCs in vitro from pluripotent stem cells has remained challenging [51]. It will therefore be interesting to consider whether we can use new HSC expansion culture conditions to “capture” HSCs generated by in vitro differentiation of pluripotent stem cells.

### How Can We Prospectively Isolate Engraftable HSCs Ex Vivo?

Prospective isolation of HSCs from ex vivo cultures remains challenging and unfortunately not all *in vivo* HSC markers are reliable ex vivo (e.g., CD38 [52] and CD49f [53] for human HSCs). In mouse HSC cultures, derived from C57BL/6 mice, transplantable HSCs are found within the CD201+c-Kit+Sca1+Lin- cell population. In single cell transplantation assays, ∼1:3 of these cells could engraft with more than 1% multilineage output in recipient mice (at 12 weeks post-transplantation) from 28-day PVA-based cultures. Expression of CD150 and the Fgd5-GFP reporter [54] and lack of CD48 [55] and CD224 [56] have also been reported to mark transplantable HSCs. For human HSCs, most work has been performed under UM171-based conditions, where HSCs are marked by CD201 [53], ITGA3 [57], and CEACAM1 [57] within the CD34+CD90+CD45RA- cell population [58]. However, these compartments only contain transplantable HSCs at an estimated frequency of 1:28, suggesting that more work is needed to identify prospective markers for ex vivo HSCs.

### How Does Ex Vivo Expansion Alter HSCs?

Another open question regards the potential penalties of ex vivo HSC expansion. Genetic mutations are thought to accumulate during cell division while telomeres shorten (in somatic telomerase-inactive tissues). Although chromosomal abnormalities or leukemic mutations have been reported to not accumulate in ex vivo cultures [27,32], further investigations are warranted. It is also currently unclear whether preleukemic mutations, such as those associated with clonal hematopoiesis [59,60,61], will preferentially expand in ex vivo cultures. There may be other cellular penalties for expansion. For example, study from Kruta et al. [62] recently demonstrated an imbalance in proteostasis in cultured HSCs. They demonstrated that HSC expansion can induce protein stress leading to upregulation of heat shock factors (Hsf1) and loss of reconstitution potential. Additionally, it will be interesting to consider how mitochondrial function is altered by extensive HSC expansion, particularly given the reported role of mitochondrial dysfunction in HSC aging [63]. Further studies are therefore warranted to better understand these ex vivo changes.

### What is the Mechanism of Action of the HSC Agonists and Mechanism of Ex Vivo Self-Renewal?

Although transplantation assays can validate that conditions expand functional HSCs ex vivo, the underlying mechanism for HSC expansion in several culture conditions still remains incompletely understood. UM171 provides a nice example of the lag time between identifying the HSC agonist (in 2014 [20]) and its target (in 2021 [22]). Whether this represents the complete mechanism of action for UM171 remains to be determined. Even less is known about why polymers, such as PVA and Soluplus, expand HSCs. Both have been shown to stabilize recombinant cytokines [28,33]. However, it is currently unclear whether they play additional roles in promoting HSC expansion. Additionally, little is known about whether HSC self-renewal is conserved in vivo and ex vivo. It is also worth considering the alternatives to ex vivo HSC expansion by self-renewal. For example, the Hoffman lab has demonstrated that cellular reprogramming using histone deacetylase inhibitors can be used to convert hematopoietic progenitors into engraftable HSCs [64]. Additionally, Gupta et al. [65] recently demonstrated that treatment of UCB units with the matricellular regulator NOV increased the frequency of long-term engraftable cells by 6-fold without increased cell division.

In summary, a number of approaches are now available to expand, maintain, and manipulate HSCs ex vivo, and the choice of culture system depends on the experimental and clinical goal. These innovations are driving exciting progress in both basic research and clinical HSCT and are opening up new research questions to address.

## Conflict of Interest Disclosure

ACW is a consultant for ImmuneBridge. The remaining authors declare no competing financial interests.
